# Effect of Crystal Symmetry of Lead Halide Perovskites on the Optical Orientation of Excitons

**DOI:** 10.1002/advs.202416782

**Published:** 2025-02-20

**Authors:** Nataliia E. Kopteva, Dmitri R. Yakovlev, Eyüp Yalcin, Ina V. Kalitukha, Ilya A. Akimov, Mikhail O. Nestoklon, Bekir Turedi, Oleh Hordiichuk, Dmitry N. Dirin, Maksym V. Kovalenko, Manfred Bayer

**Affiliations:** ^1^ Experimentelle Physik 2 Technische Universität Dortmund 44227 Dortmund Germany; ^2^ Laboratory of Inorganic Chemistry, Department of Chemistry and Applied Biosciences ETH Zürich Zürich CH‐8093 Switzerland; ^3^ Laboratory for Thin Films and Photovoltaics Empa‐Swiss Federal Laboratories for Materials Science and Technology Dübendorf CH‐8600 Switzerland; ^4^ Research Center FEMS Technische Universität Dortmund 44227 Dortmund Germany

**Keywords:** excitons, lead halide perovskites, optical spin orientation, spintronics, time‐resolved photoluminescence

## Abstract

The great variety of lead halide perovskite semiconductors represents an outstanding platform for studying crystal symmetry effects on the spin‐dependent properties. Access to them is granted through the optical orientation of exciton and carrier spins by circularly polarized photons. Here, the exciton spin polarization is investigated at 1.6 K cryogenic temperature in four lead halide perovskite crystals with different symmetries: (almost) cubic in FA_0.9_Cs_0.1_PbI_2.8_Br_0.2_ and FAPbBr_3_, and orthorhombic in MAPbI_3_ and CsPbBr_3_. Giant optical orientation of 85% is found for the excitons in FA_0.9_Cs_0.1_PbI_2.8_Br_0.2_, MAPbI_3_, and CsPbBr_3_, while it amounts to 20% in FAPbBr_3_. For all studied crystals, the optical orientation is robust to detuning of the laser photon energy from the exciton resonance, remaining constant for high energy detunings up to 0.3 eV, above which it continuously decreases to zero for detunings exceeding 1 eV. No acceleration of the spin relaxation for excitons with large kinetic energy is found in the cubic and orthorhombic crystals. This evidences the absence of the Dyakonov‐Perel spin relaxation mechanism, which is based on the Rashba‐Dresselhaus splitting of spin states at finite *k*‐vectors. This indicates that the spatial inversion symmetry is maintained in perovskite crystals, independent of the cubic or orthorhombic phase.

## Graphical abstract

Lead halide perovskite semiconductors provide a platform for studying crystal symmetry effects on spin‐dependent properties via optical orientation of exciton and carrier spins. This study examines exciton spin polarization at 1.6 K in nearly cubic and orthorhombic perovskites. Optical orientation remains robust against laser photon energy detuning, and no spin relaxation acceleration is observed for excitons with large kinetic energies. The absence of Dyakonov‐Perel spin relaxation based on the Rashba‐Dresselhaus splitting of spin states at finite *k*‐vectors suggests preserved spatial inversion symmetry, regardless of crystal phase.

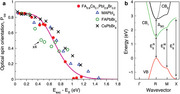

## 1 Introduction

The interest in studies of the optical properties of lead‐halide perovskites is steadily growing due to their outstanding photovoltaic^[^ ^1^ ^]^ and optoelectronic properties.^[^ ^2^, ^3^ ^]^ Their flexible chemical composition *A*Pb*X*_3_, where the cation *A* can be cesium (Cs^+^), methylammonium (MA^+^), formamidinium (FA^+^), and the anion *X* can be I^−^, Br^−^, Cl^−^, provides tunability of the bandgap from the infrared to the ultraviolet spectral range as well as temperature‐dependent crystal symmetries.^[^ ^4^ ^]^ The structural phase transition from cubic symmetry at high temperatures to orthorhombic one at cryogenic temperatures represents an excellent test bed for studying symmetry effects on spin‐dependent properties, which are important for applications in spintronics.^[^ ^3^, ^5^, ^6^, ^7^ ^]^ In turn, the spin physics sheds light on details of the crystal symmetry, as spin polarization and spin dynamics are highly susceptible to it.

Many spin‐related phenomena were explored in perovskite bulk, 2D, and nanocrystal structures. The exciton and carrier spins can be optically oriented by circularly polarized light,^[^ ^8^, ^9^, ^10^ ^]^ which is exploited in experimental techniques measuring photoluminescence polarization, time‐resolved Faraday/Kerr rotation,^[^ ^11^, ^12^ ^]^ spin‐flip Raman scattering,^[^ ^13^ ^]^ etc. The spin dynamics at cryogenic temperatures extend to nanoseconds for spin dephasing^[^ ^14^ ^]^ and to submilliseconds for longitudinal spin relaxation.^[^ ^15^ ^]^ The hyperfine interaction of excitons and carriers with the nuclear spin system is essential for the spin dynamics and relaxation,^[^ ^9^, ^14^, ^16^ ^]^ and limits the spin relaxation time in weak magnetic fields. As shown recently, the giant optical orientation of exciton spins in a FA_0.9_Cs_0.1_PbI_2.8_Br_0.2_ crystal reaches 85%,^[^ ^8^ ^]^ close to the ultimate limit of 100%. This value is robust against detuning of the laser photon energy from the exciton resonance up to 0.3 eV, providing strong experimental evidence that the Dyakonov‐Perel spin relaxation is absent and, therefore, the spin splitting in the conduction and valence bands caused by the spin‐orbit interaction is zero in this particular crystal.

In the lead halide perovskites, the spin‐orbit coupling (SOC) is large due to the heavy lead ions, which is an essential prerequisite for the spin‐dependent effects, emerging from the Rashba and Dresselhaus spin splittings of the electron and hole bands at finite wave vectors.^[^ ^17^ ^]^ The absence of spatial inversion symmetry is another key prerequisite for the SOC‐induced effects. A comprehensive review of the Rashba and Dresselhaus effects in the perovskite semiconductors, including potential origins of inversion symmetry breaking, and experimental approaches for detecting these effects can be found in refs. [18, 19]. Theoretical predictions suggest strong Rashba‐Dresselhaus splittings, but the mechanisms for symmetry breaking are still under debate. The lead halide perovskites inherently have inversion symmetry in their cubic crystal phase, and even when they transition to the tetragonal or orthorhombic phases, this inversion symmetry remains. The inversion symmetry can be broken at the crystal surface,^[^ ^20^ ^]^ at boundaries between grains, and by application of external electric or strain field.^[^ ^18^, ^21^ ^]^ For single crystals, the possible existence of ferroelectric phases has been suggested as static mechanism for Rashba‐Dresselhaus splittings.^[^ ^22^, ^23^ ^]^ For hybrid organic–inorganic perovskites, the orientation of the organic cations (MA^+^, FA^+^) and their dynamics may provide static or dynamic Rashba‐Dresselhaus effects,^[^ ^24^ ^]^ which are not expected for the fully‐inorganic perovskites with Cs^+^ cations. However, even for the fully inorganic CsPbI_3_ crystals, vibrational instabilities were predicted to give rise to dynamic distortions of the lattice.^[^ ^25^ ^]^ Given this unclear situation, unique experimental evidences are required to establish which mechanisms are relevant for which materials and for what experimental conditions.

The experiments available so far are insufficient for a comprehensive understanding of the bulk Rashba‐Dresselhaus effects. A large Rashba splitting was observed in angle‐resolved photoemission spectroscopy (ARPES) on a MAPbBr_3_ single crystal^[^ ^26^ ^]^ and by two‐photon absorption spectroscopy.^[^ ^27^ ^]^ However, the ARPES technique is sensitive only to the symmetry breaking at the surface. The presence of the surface Rashba effect on a MAPbBr_3_ single crystal was confirmed by the circular photogalvanic effect.^[^ ^28^ ^]^ Later, the absence of the static Rashba‐Dresselhaus splitting was evidenced by ARPES experiments on MAPbBr_3_ and CsPbBr_3_ crystals,^[^ ^29^ ^]^ and by second harmonic generation experiments on MAPbBr_3_ crystals.^[^ ^30^ ^]^ In MAPbI_3_ crystals, the dynamical Rashba effect caused by thermally induced structural fluctuations that break the inversion symmetry was evidenced by measuring the circular photogalvanic effect.^[^ ^31^ ^]^ The resulting spin splitting is the key basic aspect in the studied effects. Therefore, spin physics techniques, which allow one to study spin splittings and spin dynamics, can greatly contribute to understanding this still unresolved problem.

In the present paper, we study the spin orientation and relaxation of excitons to clarify the effect of the spin‐orbit coupling and identify a possible breaking of the inversion symmetry in various perovskite single crystals. We chose four lead halide perovskite crystals, FA_0.9_Cs_0.1_PbI_2.8_Br_0.2_, FAPbBr_3_, MAPbI_3_, and CsPbBr_3_ that can be distinguished by: i) different halogens (I and Br) determining the band gap energy, ii) different cations (FA^+^, MA^+^, Cs^+^), iii) hybrid organic‐inorganic and fully‐inorganic perovskites, and iv) different crystal symmetries at cryogenic temperatures ranging from almost cubic to orthorhombic. This crystal selection allows us to investigate the influence of these features on the optical orientation of exciton spins and on the exciton spin relaxation. In turn, we get information about the crystal symmetries, namely that the spatial inversion symmetry inherent to the cubic perovskite crystals is not broken in the orthorhombic crystals.

## 2 Results and Discussion

Here, we examine four lead halide perovskite crystals with bandgap energies (*E*_g_) in the visible spectral range of 1.5–2.4 eV and with different symmetries at cryogenic temperatures, namely FA_0.9_Cs_0.1_PbI_2.8_Br_0.2_, FAPbBr_3_, MAPbI_3_, and CsPbBr_3_.

### 2.1 Optical Properties

**Figure 1:**
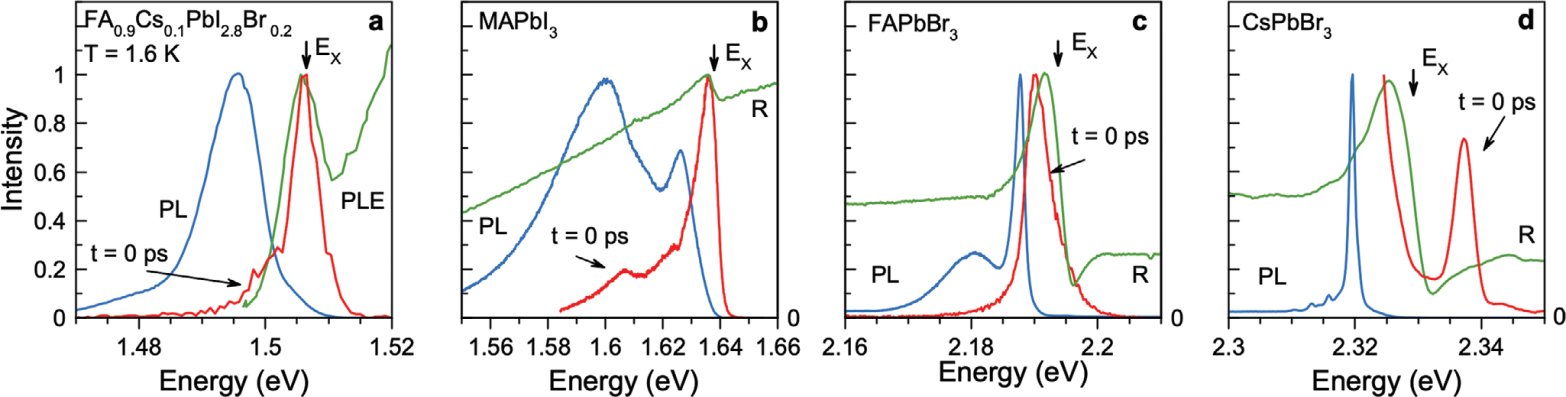
Optical properties of the studied lead halide perovskite crystals with various bandgaps at 1.6 K temperature. The blue lines show time‐integrated photoluminescence spectra measured for pulsed excitation at *E*_exc_ = 3.06 eV using the laser power density of 5 mW cm^−^^2^. The green lines show the PL excitation spectrum for FA_0.9_Cs_0.1_PbI_2.8_Br_0.2_, but reflectivity spectra for the other samples. *E*_X_ marks the exciton resonance. PL spectra right after the excitation pulse *t* = 0 ps are depicted by the red lines. Laser excitation energy is *E*_exc_ = 1.669 eV for FA_0.9_Cs_0.1_PbI_2.8_Br_0.2_, 1.771 eV for MAPbI_3_, 2.296 eV for FAPbBr_3_, and 2.385 eV for CsPbBr_3_.

The optical properties of the studied crystals at *T* = 1.6 K cryogenic temperature are collected in **Figure** **1**. The spectra of reflectivity (R) or photoluminescence excitation (the latter for FA_0.9_Cs_0.1_PbI_2.8_Br_0.2_) each show a pronounced exciton resonance, marked by an arrow and labeled with *E*_X_. Time‐integrated photoluminescence (PL) spectra shown by the blue lines demonstrate a Stokes shift of about 6 − 10 meV from the energy of the exciton resonance. The emission is contributed to bound or localized excitons and by recombination of electrons and holes separated spatially.^[^ ^8^, ^9^, ^32^, ^33^ ^]^

**Figure 2:**
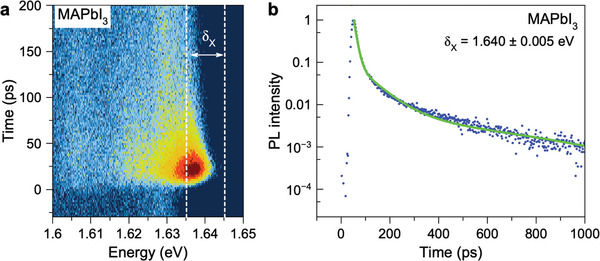
Recombination dynamics for the MAPbI_3_ crystal at *T* = 1.6 K. a) Contour plot of time‐resolved photoluminescence excited with 200 fs laser pulses at the laser photon energy of 1.771 eV. b) Recombination dynamics integrated over the δ_X_ = 1.635 − 1.645 eV spectral range around the exciton line maximum at *E*_X_ = 1.636 eV. The green line is three‐exponential fit with decay times: τ_R1_ = 15 ps, τ_R2_ = 80 ps, and τ_R3_ = 810 ps.

We are interested in excitons with a short recombination time. Their emission can be isolated spectrally and temporally by measuring time‐resolved PL using a streak camera, as shown in **Figure** **2**a for MAPbI_3_. Right after the excitation pulse action, the emission has its spectral maximum at *E*_X_ = 1.636 eV, as demonstrated in Figure 1b by the red line. The exciton dynamics spectrally integrated in the range of 1.635–1.645 eV are shown in Figure 2b. The dynamics can be fitted with a three‐exponential decay function. We assign the two short decay times of τ_R1_ = 15 ps and τ_R2_ = 80 ps to the exciton recombination. The long‐lived component with τ_R3_ = 810 ps is provided by recombination of spatially separated electrons and holes. It is a common feature for bulk perovskites that the recombination of electron‐hole pairs and of excitons is spectrally overlapping.^[^ ^9^, ^11^, ^13^, ^14^ ^]^ ref. [9] shows how these contributions can be separated by employing the Hanle and polarization recovery effects in a magnetic field.

Spectrally, the exciton emission matches the resonance in the PL excitation spectrum for FA_0.9_Cs_0.1_PbI_2.8_Br_0.2_, and in the reflectivity spectra for MAPbI_3_ and FAPbBr_3_. Its time‐integrated contribution is considerably weaker than the one of the Stokes shifted PL, so that in the time‐integrated spectra (blue lines in Figure 1), the exciton emission is not observed as separate line, but as high‐energy shoulder. In the CsPbBr_3_ crystal with a strong exciton‐polariton effect,^[^ ^34^ ^]^ a separate emission line is seen arising from the upper polariton branch at 2.3375 eV, see the red line in Figure 1d (see for details Section S1, Supporting Information).

### 2.2 Optical Orientation of Exciton Spins

The optical orientation of exciton spins induced by circularly polarized laser excitation is illustrated for the MAPbI_3_ example. Note that the MAPbI_3_ crystal has orthorhombic symmetry at *T* = 1.6 K, which is well documented by its spin‐dependent properties, including the strong anisotropy of the Landè *g*‐factors of electron and hole.^[^ ^13^, ^35^ ^]^

**Figure 3:**
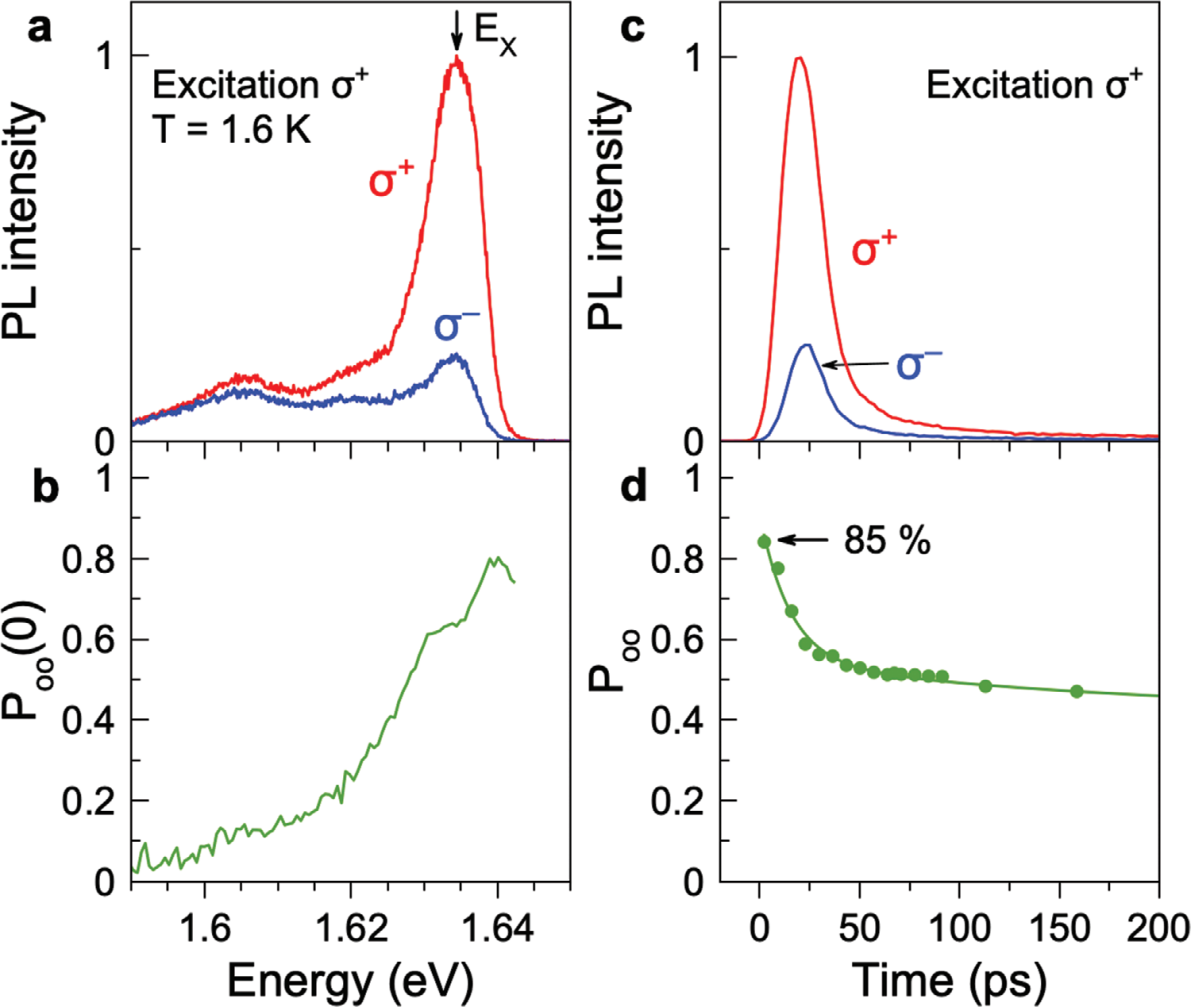
Optical orientation of excitons in MAPbI_3_ crystal. a) PL spectra right after σ^+^ polarized excitation with 200 fs laser pulses (*t* = 0 ps, *E*_exc_ = 1.698 eV, excitation density of 10 mW cm^−2^), measured in σ^+^ (red line) and σ^−^ (blue line) polarization. *T* = 1.6 K. b) Spectral dependence of the optical orientation degree calculated for the data in panel (a). c) PL dynamics measured in σ^+^ (red line) and σ^−^ (blue line) polarization for σ^+^ polarized excitation. The signal is detected in the spectral range of δ_X_ = 1.635 − 1.645 eV. d) Dynamics of the optical orientation degree *P*_oo_(*t*).

**Figure** **3**a shows the σ^+^ and σ^−^ circularly polarized PL spectra right after σ^+^ polarized excitation pulse action (*t* = 0 ps). At the exciton energy, the σ^+^ polarized emission is considerably stronger than the σ^−^ polarized one, evidencing a large degree of optical orientation. The latter we define as:

(1) $$P_{\mathrm{oo}}=\frac{I^{++}-I^{+-}}{I^{++}+I^{+-}}\;$$

here *I* ^++^ and *I* ^+−^ are the intensities of the σ^+^ and σ^−^ polarized emission measured for σ^+^ polarized excitation. The spectral dependence of *P*_oo_ calculated by Equation (1) is presented in Figure 3b. The maximal optical orientation degree of 0.85 (or 85%) is observed at the high energy flank of the exciton at 1.640 eV. Note that we reported recently the same giant optical orientation of excitons for FA_0.9_Cs_0.1_PbI_2.8_Br_0.2_ crystals^[^ ^8^ ^]^ with almost cubic crystal symmetry at cryogenic temperatures, as confirmed by isotropic carrier *g*‐factors.^[^ ^13^, ^14^ ^]^

The dynamics of the σ^+^ and σ^−^ circularly polarized exciton emission after the σ^+^ polarized pulse are shown in Figure 3c. The σ^+^ polarized dynamics have a larger amplitude than in the opposite helicity, evidencing the large optical orientation degree. The dynamics of *P*_oo_(*t*) are plotted in Figure 3d. The maximal optical orientation degree of 0.85 is observed right after the pulse action. The optical orientation decreases with an initial decay time of about 20 ps, saturating at about 0.45 for delays exceeding 200 ps, where excitons have already recombined. This behavior is similar to the recently reported optical orientation for FA_0.9_Cs_0.1_PbI_2.8_Br_0.2_ in ref. [8], where it is shown that the decrease of *P*_oo_ is not related to exciton spin relaxation, but is determined by exciton recombination. In this case, two recombination processes coexist at the same energy: fast exciton recombination and slower recombination of spatially separated electrons and holes. The exciton spin relaxation time strongly exceeds its recombination time (in FA_0.9_Cs_0.1_PbI_2.8_Br_0.2_ by a factor of 5). The observed decrease of *P*_oo_ reflects the fact that all highly‐polarized excitons have recombined, and *P*_oo_ at longer delays is contributed by the optical orientation of carriers with a smaller degree of *P*_oo_.^[^ ^9^ ^]^ Details of the time evolution of the optical orientation spectra are given in Section S2 (Supporting Information).

### 2.3 Robustness of Optical Orientation Against Optical Detuning

The surprising peculiarity of optical orientation in FA_0.9_Cs_0.1_PbI_2.8_Br_0.2_ crystals is its outstanding robustness against detuning of the excitation energy from the exciton resonance.^[^ ^8^ ^]^ This gives strong experimental evidence that the spin splitting of the conduction and valence bands at finite wave vectors, which might be provided by the Rashba and Dresselhaus effects, are absent in materials with almost cubic crystal symmetry. Therefore, it evidences that the lattice has inversion symmetry, as expected for the crystal structure of cubic perovskites.

We measured experimentally very high optical orientation of exciton emission of 85%, which is close to the ultimate limit of 100%. The ultimate limit can be achieved when the three necessary conditions for spin generation and spin relaxation are fulfilled: i) Optical matrix elements should allow photogeneration of 100% polarized electrons and carriers, even at high kinetic energies. Theoretical analysis in ref. [8] shows that this is feasible in bulk lead halide perovskites due to their band structure. Furthermore, spin polarization efficiency remains 100% even for significant energy detuning from the exciton resonance of up to 0.3 eV. ii) Photogenerated electrons and holes retain their spin polarization during energy relaxation via the emission of optical and acoustic phonons, even when tens of phonons are required to dissipate the large kinetic energy of up to 0.3 eV. It is possible in perovskites due to suppression of the Dyakonov‐Perel spin relaxation mechanisms provided by crystal symmetry. iii) Spin relaxation time of an exciton in its ground state is significantly longer than its recombination time, ensuring that spin orientation is preserved until recombination occurs.

**Figure 4:**
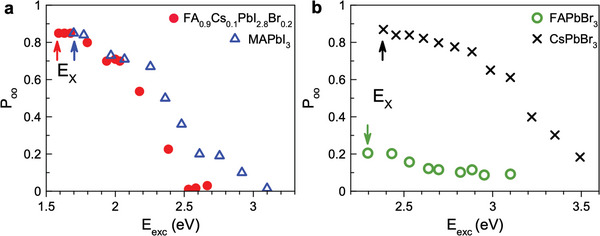
Optical orientation of excitons versus laser excitation energy. Dependence of *P*_oo_(*t* = 0) on *E*_exc_ for a) MAPbI_3_ (blue) and FA_0.9_Cs_0.1_PbI_2.8_Br_0.2_ (red), as well as b) FAPbBr_3_ (green) and CsPbBr_3_ (black). The excitation density is 10 mW cm^−2^ at *T* = 1.6 K. Arrows mark the exciton resonances (E_X_) of corresponding materials.

**Figure 5:**
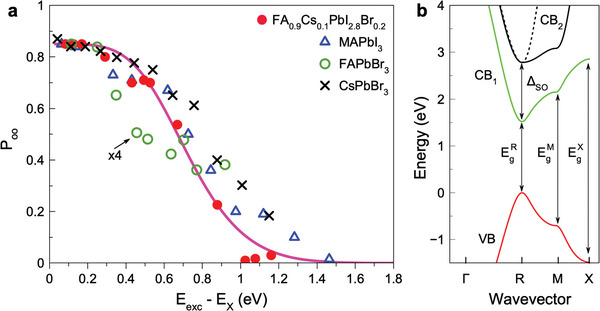
a) Optical orientation degree of excitons in various perovskite crystals as function of the optical detuning from the exciton resonance. The data are taken from Figure 4 with the exciton energy set to zero. The values for FAPbBr_3_ are multiplied by a factor of four for better comparison. The pink line is the theoretical dependence calculated with account for the Elliott‐Yafet spin relaxation due to interaction with longitudinal optical phonons, see ref. [8]. b) Calculated band diagram of bulk FA_0.9_Cs_0.1_PbI_2.8_Br_0.2_ along the Γ → *R* → *M* → *X* path. The vertical lines indicate the transition energies at the *R*, *M*, and *X* points from the valence band (VB) to the conduction band (CB_1_). The light‐ and heavy‐electron bands CB_2_ are shifted from CB_1_ by the spin‐orbit splitting (Δ_SO_).

This raises immediately the question whether the reduction of crystal symmetry to orthorhombic induces mechanisms that break the inversion symmetry. To answer, MAPbI_3_ is the optimal material as its bandgap is close to that of FA_0.9_Cs_0.1_PbI_2.8_Br_0.2_, since the gap is controlled by the iodine anions, but the crystal symmetry is reduced from cubic to orthorhombic below the phase transition temperature of 160°C. In **Figure** **4**a the dependence of the exciton optical orientation degree on the excitation energy detuned to higher values relative to the exciton resonance is shown for the MAPbI_3_ crystal by the blue triangles. *P*_oo_ starts at 0.85 for small excitation energies relative to *E*_X_ and decreases weakly up to about 0.3 eV detuning. The decrease becomes prominent for larger detunings and *P*_oo_ reaches zero for a detuning of about 1.4 eV. This behavior is similar to the findings for almost cubic FA_0.9_Cs_0.1_PbI_2.8_Br_0.2_ crystals, shown here by the red circles. As the band gaps of these materials differ by about 0.1 eV, we plot for better comparison the data in **Figure** **5**a as function of the detuning energy from the exciton resonance (*E*_exc_ − *E*_X_), so that *E*_X_ = 0. The dependence for the two materials coincide remarkably well with each other. From these data, we conclude that the reduction of crystal symmetry from almost cubic to orthorhombic does not result in activation of any additional spin relaxation mechanisms. The main candidate would be the Dyakonov‐Perel mechanism which should become efficient due to a spin splitting of the electron and hole states at finite wave vectors in crystals without inversion symmetry combined with a strong spin‐orbit interaction. Therefore, one can conclude that the spatial inversion symmetry is maintained for the orthorhombic MAPbI_3_ crystal. Experimental studies in refs. [36, 37] show that the Dyakonov‐Perel mechanism reduces optical orientation at small optical detunings and accelerates spin relaxation in conventional III‐V and II‐VI semiconductors.

To collect comprehensive information on the whole class of lead halide perovskite semiconductors, we examine experimentally two more materials with bromine anions resulting in larger bandgaps. These are the almost cubic FAPbBr_3_ and the orthorhombic CsPbBr_3_ crystals. Their optical spectra are shown in Figure 1c,d. In the first material, the electron and hole *g*‐factors are almost isotropic,^[^ ^38^ ^]^ and in the second one the *g*‐factors show a pronounced anisotropy.^[^ ^13^ ^]^ The optical orientation of excitons in these crystals measured at various excitation energies is shown in Figure 4b. For small detunings from *E*_X_, *P*_oo_(*t* = 0) reaches 0.85 in CsPbBr_3_ and 0.20 in FAPbBr_3_. Despite the difference in the absolute values, their dependence on excitation energy is very similar, as can be better seen in Figure 5a, where the data for FAPbBr_3_ are multiplied by a factor of four to be normalized to 0.85 value in CsPbBr_3_. The basically identical dependence on the detuning allows us to conclude that the difference in the optical orientation degree is provided by a more efficient spin relaxation of cold excitons in FAPbBr_3_. We suggest that the responsible mechanism is provided by exciton interaction with the nuclear spin fluctuations,^[^ ^9^, ^16^ ^]^ which requires, however, further investigations beyond the scope of the present study.

In Figure 5a the exciton optical orientation in the four studied materials is compared. All of them show a similar dependence on the excitation energy detuning, which evidences that the dependence is determined by the band structure of the lead halide perovskites around the bandgap to energies 0.5 − 0.8 eV higher than the conduction and valence band extrema. Here, we keep in mind that the effective masses of electrons and holes in the lead halide perovskites are about equal to each other. Note that the investigated excitation energy detuning is smaller than the spin‐orbit‐splitting Δ_SO_ to the heavy‐ and light‐electron bands of about 1.2–1.5 eV and the energy gap at the M‐point, which exceeds the bandgap at the R‐point by about 1.33 eV, see Figure 5b. The depolarization at higher excitation energies can be attributed to: i) The deviations from strict selection rules due to detuning from the R point of Brillouin zone. For even larger detunings (>1.3 eV), transitions to the spin‐off conduction band, CB_2_, may further reduce the polarization. ii) Spin‐flip scattering during carrier energy relaxation on optical phonons contributes through the Elliott‐Yafet spin relaxation mechanism.

### 2.4 Discussion

The optical orientation degree is determined by two factors: i) the efficiency of spin initialization induced by circularly polarized photons for the interband optical transition and ii) the loss of spin polarization during the exciton lifetime τ_R_. The latter is controlled by the factor τ_s_/(τ_s_ + τ_R_) that reduces the initial optical orientation, where τ_s_ is the exciton spin relaxation time. The band structure of lead halide perovskites is favorable for obtaining high optical orientation close to the ultimate limit of 100% .^[^ ^8^ ^]^ In the vicinity of the bandgap, i.e., in the detuning range up to about 0.3 eV, the spins can be optically oriented with 100% efficiency. Also, due to the presence of inversion symmetry of the crystal lattice, spin splittings of the bands through the spin‐orbit interaction are absent, suppressing the spin relaxation by the Dyakonov‐Perel mechanism. Instead, the spin relaxation is governed by the rather inefficient Elliott‐Yafet mechanism and the electron‐hole exchange interaction in the exciton. These expectations are in agreement with the observed high optical orientation degree of 0.85 measured for three of the studied materials. We can safely conclude that τ_s_ ≫ τ_R_ for the excitons in MAPbI_3_, FA_0.9_Cs_0.1_PbI_2.8_Br_0.2_, and CsPbBr_3_.

An in‐depth theoretical analysis based on tight‐binding calculations of the spin initialization efficiency at large detunings has been performed in ref. [8]. The line in Figure 5a shows the calculated dependence, which matches well the experimental data for all four materials. The analysis shows that the decrease of optical orientation at larger detunings originates from the deviation of the optical helicity selection rules from the strict ones at the R‐point. Also, additional depolarization comes from the spin‐flip scattering of excitons during the energy relaxation provided by the Elliott‐Yafet mechanism. It is well established that CsPbBr_3_ at low temperatures has an orthorhombic crystal structure with space group #62. This group has an inversion center, so that no spin splitting in the bands is expected. However, samples with organic cations (MA^+^ and FA^+^) may show a lower local symmetry due to the oriented organic cations.^[^ ^19^ ^]^ Close similarities of spin properties found for Cs, MA, and FA‐based materials let us conclude that the latter effect does not contribute to spin dynamics.

We emphasize that the experimental technique used in our study delivers information about the exciton properties inside a crystal and not at its surface, where excitons are typically destroyed. Therefore, our experiments cannot detect a possible breaking of the inversion symmetry within the few monolayers close to the crystal surface, as reported recently, e.g., for a MAPbBr_3_ single crystal by the circular photogalvanic effect.^[^ ^28^ ^]^

Finally, we discuss the impact of optical orientation of carriers on the observed effects. The presence of resident carriers can lead to the formation of trions. However, trions form at the band edges (zero momentum), where the Dyakonov‐Perel mechanism is suppressed. At higher kinetic energies, the trion formation is not possible. Carrier spin relaxation may be governed by the Elliott‐Yafet and Bir‐Aronov‐Pikus mechanisms. The Elliott‐Yafet mechanism, however, is suppressed for localized carriers with zero momentum. The Bir‐Aronov‐Pikus mechanism requires high density of carriers to facilitate their scattering. In this study, we employ a low excitation density of 10 mW cm^−^^2^, resulting in a modest electron‐hole pair density of 10^13^ cm^−3^. These spin relaxation mechanisms may become significant for delocalized carriers in experiments conducted at elevated temperatures.

## 3 Conclusion

We have demonstrated that the exciton spins can be optically oriented in FA_0.9_Cs_0.1_PbI_2.8_Br_0.2_, MAPbI_3_, CsPbBr_3_, and FAPbBr_3_ crystals and in most of them a very high degree of optical orientation of 85% is reached at cryogenic temperatures. For different perovskite compositions and symmetries, the optical orientation is robust against detuning of the laser excitation energy from the exciton resonance. We find that the exciton spin relaxation mechanisms are not influenced by the symmetry reduction from almost cubic to orthorhombic, evidencing that the spatial inversion symmetry inherent to cubic perovskite crystals is not broken for orthorhombic crystals.

In bulk perovskite crystals, the inversion symmetry may be absent at their surface, and in polycrystalline films, it may get lost at the crystalline boundaries. Also, external factors, like axial strain and electric field, may break the inversion symmetry. The surface effect may become detrimental for perovskite nanocrystals and 2D materials, where a Rashba spin splitting has been reported, based on experimental data^[^ ^27^, ^39^, ^40^ ^]^ and theoretical predictions.^[^ ^40^, ^41^ ^]^ We are convinced that the optical orientation technique is an extremely useful tool for examining the effects associated with the crystal symmetry in perovskite nanostructures.

## 4 Experimental Section

### Samples

Lead halide perovskite single crystals were studied with different bandgaps. The crystals were grown out of solution using the inverse temperature crystallization (ITC) technique.^[^ ^42^, ^43^, ^44^ ^]^ For the FA_0.9_Cs_0.1_PbI_2.8_Br_0.2_, FAPbBr_3_, and CsPbBr_3_ crystals studied here, details of the synthesis are given in ref. [13]. The MAPbI_3_ crystal was synthesized from PbI_2_ and MAI perovskite precursors between two polytetrafluoroethylene coated glasses, following ref. [45] The sample had a square shape of 1 × 1 mm^2^ area in the (001) crystallographic plane with a thickness of 30 µm.

The hybrid organic–inorganic compounds FA_0.9_Cs_0.1_PbI_2.8_Br_0.2_ (*E*_g_ = 1.520 eV) and MAPbI_3_ (1.652 eV) had bandgap energies close to the near‐infrared at *T* = 1.6 K temperature. Replacing the iodine halogen with bromine results in a blue shift of the bandgap for FAPbBr_3_ (2.216 eV). To develop a complete picture, the fully inorganic perovskite CsPbBr_3_ (2.359 eV) was also studied. The *g*‐factor tensor of electrons and holes measured at the cryogenic temperatures was isotropic for FA_0.9_Cs_0.1_PbI_2.8_Br_0.2_ and FAPbBr_3_, reflecting their almost cubic crystal symmetry.^[^ ^13^ ^]^ Change of the cation from FA^+^ to MA^+^ and Cs^+^ leads to the symmetry reduction, evidenced by a strong *g*‐factor anisotropy of carriers.^[^ ^13^ ^]^

### Optical Measurements

For the low‐temperature optical measurements, a liquid helium cryostat was used with the temperature variable from 1.6 K up to 300 K. At *T* = 1.6 K, the sample was immersed in superfluid helium.

### Photoluminescence and Reflectivity Measurements

The time‐integrated photoluminescence (PL) and reflectivity spectra were measured with a 0.5 m spectrometer equipped with a charge‐coupled‐device (CCD) camera. A halogen lamp was used as light source for the reflectivity measurements.

### Time‐Resolved Photoluminescence

The spectrally‐resolved PL dynamics were measured using a spectrometer with a 300 grooves/mm diffraction grating and a streak camera with 10 ps time resolution. Laser pulses with 200 fs duration and central photon energies from 1.7 eV (730 nm) to 3.5 eV (350 nm) taken from a tunable Coherent Chameleon Discovery laser (repetition rate of 80 MHz) with a second harmonic generation unit were used for PL excitation. To study the effect of optical orientation, circular polarized (σ^+^/σ^−^) excitation light was used, and subsequently the circular polarization of the emission was analyzed.

## Conflict of Interest

The authors declare no conflict of interest.

## Supporting information

Supporting Information

## Data Availability

The data that support the findings of this study are available from the corresponding author upon reasonable request.

## References

[1] A. K. Jena , A. Kulkarni , T. Miyasaka , Chem. Rev. 2019, 5, 3036. 10.1021/acs.chemrev.8b00539 30821144

[2] Halide Perovskites for Photonics (eds. A. Vinattieri and G. Giorgi ), AIP Publishing, Melville, New York 2021.

[3] Hybrid Organic Inorganic Perovskites: Physical Properties and Applications (eds. Z. V. Vardeny and M. C. Beard ), World Scientific, Singapore 2022.

[4] M. Simenas , A. Gagor , J. Banys , M. Maczka , Chem. Rev. 2024, 124, 2281. 38421808 10.1021/acs.chemrev.3c00532 PMC10941198

[5] J. Wang , C. Zhang , H. Liu , R. McLaughlin , Y. Zhai , S. R. Vardeny , X. Liu , S. McGill , D. Semenov , H. Guo , R. Tsuchikawa , V. V. Deshpande , D. Sun , Z. V. Vardeny , Nat. Commun. 2019, 10, 129. 30631053 10.1038/s41467-018-07952-x PMC6328620

[6] W. Ning , J. Bao , Y. Puttisong , F. Moro , L. Kobera , S. Shimono , L. Wang , F. Ji , M. Cuartero , S. Kawaguchi , S. Abbrent , H. Ishibashi , R. De Marco , I. A. Bouianova , G. A. Crespo , Y. Kubota , J. Brus , D. Y. Chung , L. Sun , W. M. Chen , M. G. Kanatzidis , F. Gao , Sci. Adv. 2020, 6, eabb5381. 33158858 10.1126/sciadv.abb5381 PMC7673701

[7] Y.‐H. Kim , Y. Zhai , H. Lu , X. Pan , C. Xiao , E. A. Gaulding , S. P. Harvey , J. J. Berry , Z. V. Vardeny , J. M. Luther , M. C. Beard , Science 2021, 371, 1129. 33707260 10.1126/science.abf5291

[8] N. E. Kopteva , D. R. Yakovlev , E. Yalcin , I. A. Akimov , M. O. Nestoklon , M. M. Glazov , M. Kotur , D. Kudlacik , E. A. Zhukov , E. Kirstein , O. Hordiichuk , D. N. Dirin , M. V. Kovalenko , M. Bayer , Adv. Sci. 2024, 11, 2403691. 10.1002/advs.202403691 PMC11336922 38884160

[9] D. Kudlacik , N. E. Kopteva , M. Kotur , D. R. Yakovlev , K. V. Kavokin , C. Harkort , M. Karzel , E. A. Zhukov , E. Evers , V. V. Belykh , M. Bayer , ACS Photonics 2024, 11, 2757.

[10] D. Giovanni , H. Ma , J. Chua , M. Grätzel , R. Ramesh , S. Mhaisalkar , N. Mathews , T. Ch. Sum , Nano Lett. 2015, 15, 1553. 25646561 10.1021/nl5039314

[11] V. V. Belykh , D. R. Yakovlev , M. M. Glazov , P. S. Grigoryev , M. Hussain , J. Rautert , D. N. Dirin , M. V. Kovalenko , M. Bayer , Nat. Commun. 2019, 10, 673. 30737402 10.1038/s41467-019-08625-z PMC6368575

[12] P. Odenthal , W. Talmadge , N. Gundlach , R. Wang , C. Zhang , D. Sun , Z.‐G. Yu , V. Z. Vardeny , Y. S. Li , Nat. Phys. 2017, 13, 894.

[13] E. Kirstein , D. R. Yakovlev , M. M. Glazov , E. A. Zhukov , D. Kudlacik , I. V. Kalitukha , V. F. Sapega , G. S. Dimitriev , M. A. Semina , M. O. Nestoklon , E. L. Ivchenko , N. E. Kopteva , D. N. Dirin , O. Nazarenko , M. V. Kovalenko , A. Baumann , J. Höcker , V. Dyakonov , M. Bayer , Nat. Commun. 2022, 13, 3062. 35654813 10.1038/s41467-022-30701-0 PMC9163162

[14] E. Kirstein , D. R. Yakovlev , M. M. Glazov , E. Evers , E. A. Zhukov , V. V. Belykh , N. E. Kopteva , D. Kudlacik , O. Nazarenko , D. N. Dirin , M. V. Kovalenko , M. Bayer , Adv. Mater. 2022, 34, 2105263. 34606138 10.1002/adma.202105263 PMC11468769

[15] V. V. Belykh , M. L. Skorikov , E. V. Kulebyakina , E. V. Kolobkova , M. S. Kuznetsova , M. M. Glazov , D. R. Yakovlev , Nano Lett. 2022, 22, 4583. 35621509 10.1021/acs.nanolett.2c01673

[16] E. Kirstein , D. S. Smirnov , E. A. Zhukov , D. R. Yakovlev , N. E. Kopteva , D. N. Dirin , O. Hordiichuk , M. V. Kovalenko , M. Bayer , Nat. Commun. 2023, 14, 6683. 37865649 10.1038/s41467-023-42265-8 PMC10590392

[17] A. Manchon , H. C. Koo , J. Nitta , S. M. Frolov , R. A. Duine , Nat. Mater. 2015, 14, 871. 26288976 10.1038/nmat4360

[18] M. Kepenekian , R. Robles , C. Katan , D. Sapori , L. Pedesseau , J. Even , ACS Nano 2015, 9, 11557. 26348023 10.1021/acsnano.5b04409

[19] M. Kepenekian , J. Even , J. Phys. Chem. Lett. 2017, 8, 3362. 28661150 10.1021/acs.jpclett.7b01015

[20] E. Mosconi , T. Etienne , F. De Angelis , J. Phys. Chem. Lett. 2017, 8, 2247. 28467716 10.1021/acs.jpclett.7b00328

[21] L. Leppert , S. E. Reyes‐Lillo , J. B. Neaton , J. Phys. Chem. Lett. 2016, 7, 3683. 27577732 10.1021/acs.jpclett.6b01794

[22] M. Kim , J. Im , A. J. Freeman , J. Ihm , H. Jin , PNAS 2014, 111, 6900. 24785294 10.1073/pnas.1405780111 PMC4024856

[23] Zh.‐G. Yu , Y. S. Li , J. Phys. Chem. C 2019, 123, 14701.

[24] A. Marronnier , G. Roma , M. A. Carignano , Y. Bonnassieux , C. Katan , J. Even , E. Mosconi , F. De Angelis , J. Phys. Chem. C 2019, 123, 291.

[25] S. McKechnie , J. M. Frost , D. Pashov , P. Azarhoosh , A. Walsh , M. van Schilfgaarde , Phys. Rev. B 2018, 98, 085108.

[26] D. Niesner , M. Wilhelm , I. Levchuk , A. Osvet , S. Shrestha , M. Batentschuk , C. Brabec , T. Fauster , Phys. Rev. Lett. 2016, 117, 126401. 27689285 10.1103/PhysRevLett.117.126401

[27] E. Lafalce , E. Amerling , Z.‐G. Yu , P. C. Sercel , L. Whittaker‐Brooks , Z. V. Vardeny , Nat. Commun. 2022, 13, 483. 35078984 10.1038/s41467-022-28127-9 PMC8789784

[28] Z. Huang , Sh. R. Vardeny , T. Wang , Z. Ahmad , A. Chanana , E. Vetter , Sh. Yang , X. Liu , G. Galli , A. Amassian , Z. V. Vardeny , D. Sun , Appl. Phys. Rev. 2021, 8, 031408.

[29] M. Sajedi , M. Krivenkov , D. Marchenko , A. Varykhalov , J. Sánchez‐Barriga , E. D. L. Rienks , O. Rader , Phys. Rev. B 2020, 102, 081116.

[30] K. Frohna , T. Deshpande , J. Harter , W. Peng , B. A. Barker , J. B. Neaton , S. G. Louie , O. M. Bark , D. Hsieh , M. Bernardi , Nat. Commun. 2018, 9, 1829. 29739939 10.1038/s41467-018-04212-w PMC5940805

[31] D. Niesner , M. Hauck , Sh. Shrestha , I. Levchukc , G. J. Matt , A. Osvet , M. Batentschuk , Ch. Brabec , H. B. Weber , Th. Fauster , PNAS 2018, 115, 9509. 30181293 10.1073/pnas.1805422115 PMC6156630

[32] A. D. Wright , R. L. Milot , G. E. Eperon , H. J. Snaith , M. B. Johnston , L. M. Herz , Adv. Funct. Mater. 2017, 27, 1700860.

[33] D. W. deQuilettes , K. Frohna , D. Emin , T. Kirchartz , V. Bulovic , D. S. Ginger , S. D. Stranks , Chem. Rev. 2019, 119, 11007. 31496228 10.1021/acs.chemrev.9b00169

[34] D. R. Yakovlev , S. A. Crooker , M. A. Semina , J. Rautert , J. Mund , D. N. Dirin , M. V. Kovalenko , M. Bayer , Phys. Stat. Sol. RRL 2024, 18, 2300407.

[35] E. Kirstein , D. R. Yakovlev , E. A. Zhukov , J. Höcker , V. Dyakonov , M. Bayer , ACS Photonics 2022, 9, 1375.

[36] R. R. Parsons , Canadian Journal of Physics 1971, 49, 1850.

[37] A. I. Ekimov , V. I. Safarov , JETP Lett. 1971, 13, 495.

[38] E. Kirstein , E. A. Zhukov , D. R. Yakovlev , N. E. Kopteva , E. Yalcin , I. A. Akimov , O. Hordiichuk , D. N. Dirin , M. V. Kovalenko , M. Bayer , J. Phys. Chem. Lett. 2024, 15, 2893. 38448798 10.1021/acs.jpclett.4c00098

[39] Y. Zhai , S. Baniya , Ch. Zhang , J. Li , P. Haney , Ch.‐X. Sheng , E. Ehrenfreund , Z. V. Vardeny , Science Advances 2017, 3, e1700704. 28782030 10.1126/sciadv.1700704 PMC5533538

[40] M. A. Becker , R. Vaxenburg , G. Nedelcu , P. C. Sercel , A. Shabaev , M. J. Mehl , J. G. Michopoulos , S. G. Lambrakos , N. Bernstein , J. L. Lyons , Th. Stöferle , R. F. Mahrt , M. V. Kovalenko , D. J. Norris , G. Rainó , Al. L. Efros , Nature 2018, 553, 189. 29323292 10.1038/nature25147

[41] P. C. Sercel , Al. L. Efros , Phys. Rev. B 2023, 107, 195436.

[42] D. N. Dirin , I. Cherniukh , S. Yakunin , Y. Shynkarenko , M. V. Kovalenko , Chemistry of Materials 2016, 28, 8470. 29430079 10.1021/acs.chemmater.6b04298 PMC5805401

[43] J. Höcker , F. Brust , M. Armer , V. Dyakonov , Cryst. Eng. Comm. 2021, 23, 2202.

[44] O. Nazarenko , S. Yakunin , V. Morad , I. Cherniukh , M. V. Kovalenko , NPG Asia Mater. 2017, 9, e373.

[45] Ch. Yang , J. Yin , H. Li , Kh. Almasabi , L. Gutiérrez‐Arzaluz , I. Gereige , J.‐L. Brédas , O. M. Bakr , O. F. Mohammed , ACS Energy Lett. 2022, 7, 1544.

