# Supplementary material for: Effect of Crystal Symmetry of Lead Halide Perovskites on the Optical Orientation of Excitons
Source: Adv Sci (Weinh). 2025 Feb 20;12(15):2416782. doi: 10.1002/advs.202416782 (PMC12005751; doi:10.1002/advs.202416782)
Supplement: Supplementary file 1 — Supporting Information [file ADVS-12-2416782-s001.pdf]

ADVANCED SCIENCE
Open Access

Supporting Information

for *Adv. Sci.*, DOI 10.1002/advs.202416782

Effect of Crystal Symmetry of Lead Halide Perovskites on the Optical Orientation of Excitons

Nataliia E. Kopteva, Dmitri R. Yakovlev*, Eyüp Yalcin, Ina V. Kalitukha, Ilya A. Akimov, Mikhail O. Nestoklon, Bekir Turedi, Oleh Hordiichuk, Dmitry N. Dirin, Maksym V. Kovalenko and Manfred Bayer*

Supporting Information:
Effect of crystal symmetry of lead halide perovskites on the optical orientation of excitons

Nataliia E. Kopteva[1], Dmitri R. Yakovlev[1], Eyüp Yalcin[1], Ina V. Kalitukha[1], Ilya A. Akimov[1], Mikhail O. Nestoklon[1], Bekir Türedi[2,3], Oleh Hordiichuk[2,3], Dmitry N. Dirin[2,3], Maksym V. Kovalenko[2,3], and Manfred Bayer[1]

[1]*Experimentelle Physik 2, Technische Universität Dortmund, 44227 Dortmund, Germany*

[2]*Laboratory of Inorganic Chemistry, Department of Chemistry and Applied Biosciences, ETH Zürich, CH-8093 Zürich, Switzerland and*

[3]*Laboratory for Thin Films and Photovoltaics, Empa-Swiss Federal Laboratories for Materials Science and Technology, CH-8600 Dübendorf, Switzerland*

(Dated: January 15, 2025)

S1. EXCITON-POLARITON EFFECT IN $CsPbBr_3$

Figure S1(a) presents the time-resolved and spectrally-resolved photoluminescence in $CsPbBr_3$ crystal, demonstrating the exciton-polariton formation[S1]. The spectra show emission from the upper (UPB) and lower (LPB) polariton branches.

To obtain the PL dynamics, we integrated the signal within specific spectral ranges, marked in Figure S1(a) by $\delta_{\rm UPB} = 2.337 \pm 0.003$ eV and $\delta_{\rm LPB} = 2.326 \pm 0.003$ eV for the upper and lower polariton branches. The integrated dynamics are shown in Figure S1(b). The blue data points are the UPB and the red ones for the LPB. The dynamics are fitted by a two-exponential decay function. The decay times for the UPB are 65 ps and 280 ps, while for the LPB are 30 ps and 330 ps.

Optical orientation of exciton spins reaches its maximum right after the optical pulse action for both polariton branches. Figure S1(d) illustrates the spectral dependence of optical orientation calculated for σ^+ and σ^- polarized spectra measure under σ^+ polarized excitation, see Figure S1(c). $P_{\rm oo}$ spectral dependence has maximum of 0.65 at the UPB, it decreases at lower energies reaching about 0.40 at the LPB.

Figure S1(e) shows the dependence of the optical orientation degree on the excitation energy (bottom axis) and detuning from the exciton resonance (top axis). Both polariton branches exhibit a similar trend characterized by robustness to optical detuning. However, the overall degree of orientation on the LPB is smaller compared to the UPB due to the specifics of the exciton energy relaxation via acoustic phonons.

S2. TIME EVOLUTION OF PHOTOLUMINESCENCE AND OPTICAL ORIENTATION SPECTRA

Figures S2(a) and S2(d) present normalized photoluminescence (PL) spectra detected at various time delays for $MAPbI_3$ and $FA_{0.9}Cs_{0.1}PbI_{2.8}Br_{0.2}$ crystals. For both samples, the temporal evolution of the spectra exhibits similar behavior. Immediately after the pulse arrival the exciton photoluminescence dominates in the spectra, as shown by the red curves in Figures S2(a) and S2(d). With increasing time delay after the excitation pulse the PL peak shifts to lower energies and decreases in amplitude. At a delay of 200 ps the recombination of spatially separated electron-hole pairs becomes the dominant process, forming a low-energy PL line (see black curves in Figures S2(a) and S2(d)). The maximal intensity of the photoluminescence is shown in Figures S2(b) and S2(e).

The time evolution of the optical orientation degree is shown in Figures S2(c) and S2(f) for the $MAPbI_3$ and $FA_{0.9}Cs_{0.1}PbI_{2.8}Br_{0.2}$ crystals. For both samples, the temporal evolution of the $P_{\rm oo}$ spectra exhibits similar behavior. Right after the pulse action $P_{\rm oo}$ has the maximum on the exciton spectral position, reaching the maximum value of 0.85. Over time, $P_{\rm oo}$ decreases reaching the value typical for the carrier optical orientation. At the low energies, the opticla orientation degree is contributed by the electron-hole pairs reaching values of 0.25 for $MAPbI_3$ and 0.35 for $FA_{0.9}Cs_{0.1}PbI_{2.8}Br_{0.2}$.

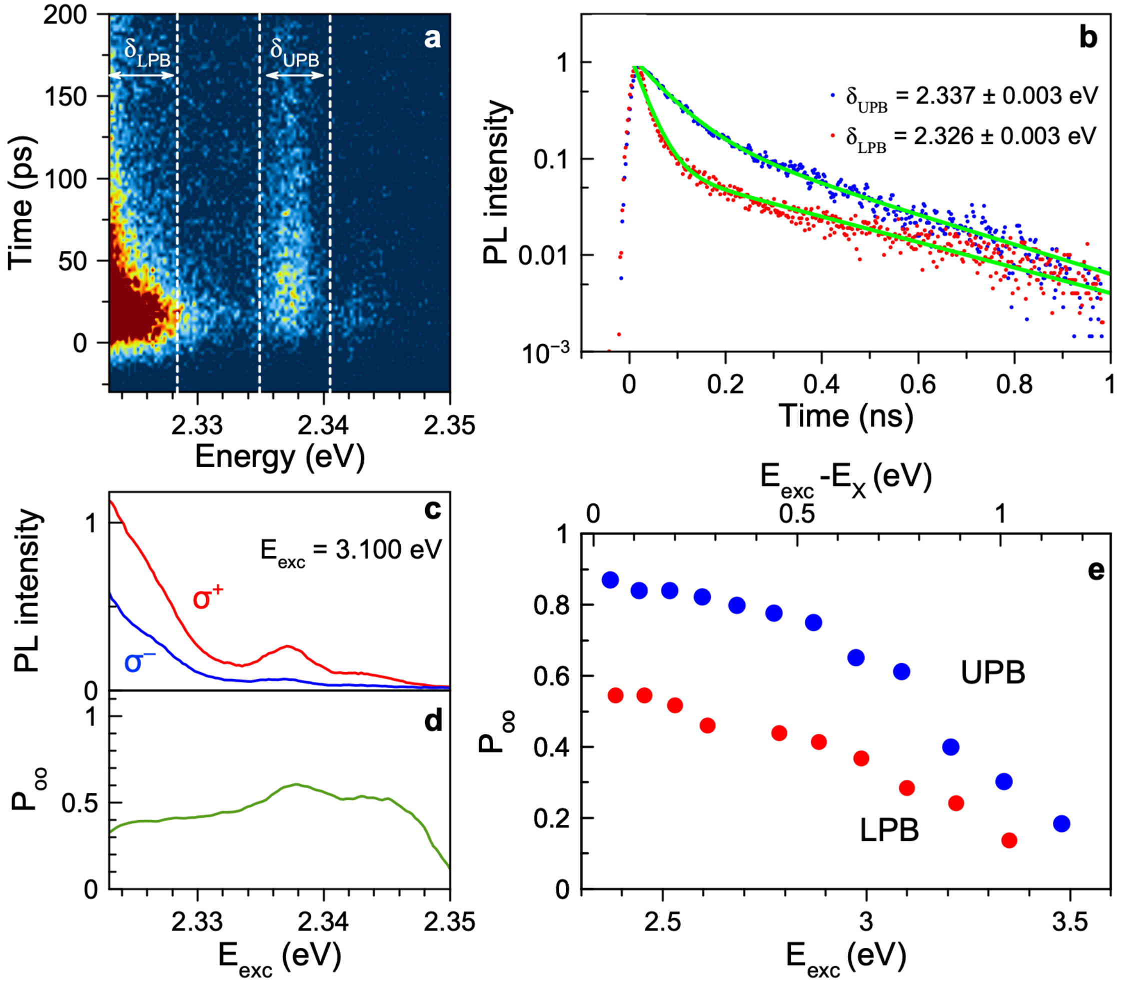

FIG S1. Exciton-polariton effect in $CsPbBr_3$ measured at $T = 1.6$ K. (a) Contour plot of time-resolved photoluminescence excited with 200 fs laser pulses at the laser photon energy of 2.385 eV and excitation density of 10 $\mathrm{mW/cm^2}$. (b) Recombination dynamics integrated over the $\delta_{\mathrm{UPB}} = 2.337 \pm 0.003$ eV (blue) and $\delta_{\mathrm{LPB}} = 2.326 \pm 0.003$ eV (red) spectral ranges characteristic for the upper polariton branch and lower polariton branch, respectively. The green lines are the two-exponential fit with parameters given in text. (c) Circularly polarized PL spectra measured right after σ^+ polarized excitation at $E_{\mathrm{exc}} = 3.100$ eV. (d) Spectral dependence of the optical orientation degree calculated for panel (c) data. (e) Optical orientation of excitons on UPB (blue) and LPB (red) in dependence on the laser excitation energy.

REFERENCES

[S1] D. R. Yakovlev, S. A. Crooker, M. A. Semina, J. Rautert, J. Mund, D. N. Dirin, M. V. Kovalenko, M. Bayer, Exciton–polaritons in $CsPbBr_3$ crystals revealed by optical reflectivity in high magnetic fields and two-photon spectroscopy. *Phys. Stat. Sol. RRL* **2024**, *18*, 2300407.

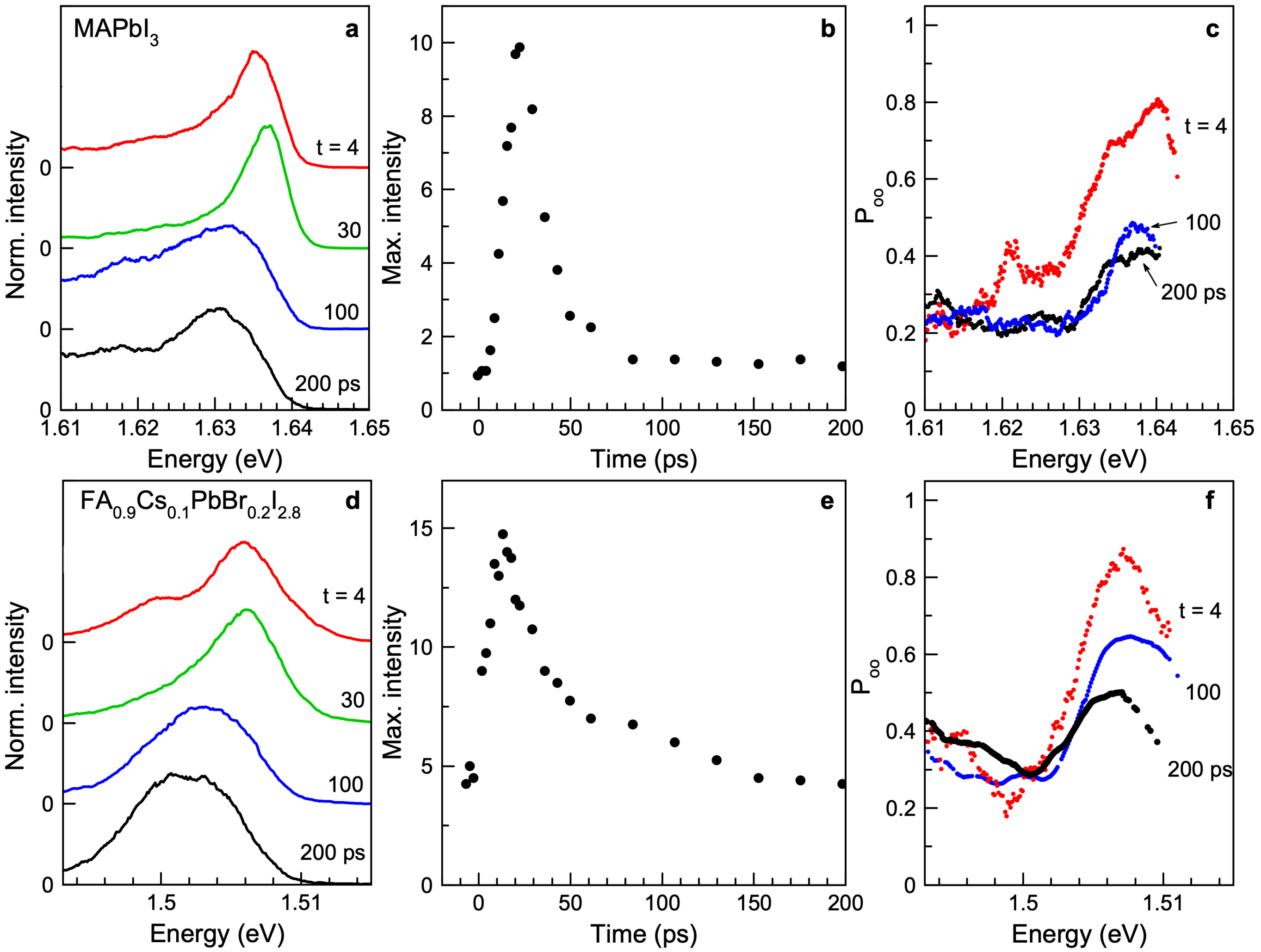

FIG S2. Time evolution of photoluminescence and optical orientation spectra in $MAPbI_3$ and $FA_{0.9}Cs_{0.1}PbI_{2.8}Br_{0.2}$ crystals at $T = 1.6$ K. (a) Normalized photoluminescence spectra detected at $t = 4$ ps (red), 30 ps (green), 100 ps (blue), and 200 ps (black) for $MAPbI_3$. $E_{\rm exc} = 1.771$ eV and excitation density is 10 mW/cm^2. (b) Maximal intensity of the PL spectra in dependence on time. (c) Spectral dependence of optical orientation degree detected at $t = 4$ ps (red), 100 ps (blue), and 200 ps (black). (d) Normalized photoluminescence spectra detected at $t = 4$ ps (red), 30 ps (green), 100 ps (blue), and 200 ps (black) for $FA_{0.9}Cs_{0.1}PbI_{2.8}Br_{0.2}$. $E_{\rm exc} = 1.669$ eV and excitation density is 10 mW/cm^2. (e) Maximal intensity of the PL spectra in dependence on time. (e) Spectral dependence of optical orientation degree detected at $t = 4$ ps (red), 100 ps (blue), and 200 ps (black).
